# Older Canadians’ Identity and Well-Being in Retirement

**DOI:** 10.1177/00914150211001586

**Published:** 2021-03-19

**Authors:** Nicky J. Newton

**Affiliations:** 1 8431 Wilfrid Laurier University, Waterloo, Canada

**Keywords:** identity processes, unplanned retirement, hedonic and eudaimonic well-being

## Abstract

Retirement can be a time of identity disruption for many older adults. Identity process theory (Whitbourne et al., 2002) states that age-related changes, such as retirement, can prompt an individual to incorporate new information about themselves into their personal identity using one of three identity process: assimilation, accommodation, and balance. Additionally, individual identity and the manner in which individuals retire—voluntary or involuntary—are associated with post-retirement well-being (Newton et al., 2018). The current study examined the relationship between identity processes, planned/unplanned retirement, and hedonic (life satisfaction) and eudaimonic (meaning in life) well-being in a sample of retired Canadians. Results indicated that identity accommodation and balance were associated with both types of post-retirement well-being, whereas unplanned retirement was consistently only related to life satisfaction. This study emphasizes the importance of including individual difference factors when examining older adults’ well-being and the utility of measuring well-being in multiple ways.

## Older Canadians’ Identity and Post-Retirement Well-Being

Well-being in adulthood follows a U-shaped curve (e.g., Blanchflower & Oswald, 2008; Stone et al., 2010); that is, following a decrease from young to middle adulthood, well-being increases for midlife and older adults. However, the antecedents and correlates of the U-shaped curve are still being documented, with retirement a common marker of change. In some studies, psychological well-being in later life increases at or after retiring (Kim & Moen, 2001; Latif, 2011), whereas in other studies, well-being decreases (Dingemans & Henkens, 2015). Research also supports the notion that the experience of retirement is individual, with acknowledgment of the need to examine a wider range of personal resource factors (Shultz & Wang, 2011; Wong & Earl, 2009), such as personality (Ryan et al., 2017) or personal identity (Newton & Ottley, 2020). Additionally, the context of retirement—for instance, the amount of control one has in choosing when and how to retire—can have important consequences for post-retirement well-being (Calvo et al., 2009; Quine et al., 2007).

The present study uses quantitative and qualitative data to examine identity and well-being in the post-retirement period; specifically, how the context in which retirement occurred (voluntary vs. involuntary) as well as an individual’s identity process are associated with hedonic and eudaimonic well-being. This study is novel: it measures well-being in two ways, using a sample of Canadian retirees, and contributes further information to our understanding of the antecedents and correlates of the U-shaped curve of well-being.

### Theoretical Framework

In this study, the life course perspective (Elder, 1985, 1995; Settersten, 2003) provides the basis for examining identity and well-being in the post-retirement period. According to this perspective, how a person experiences transitions, such as retirement, is related to their personal attributes (e.g., identity) as well as more contextual factors, such as the characteristics of the job, their attachment to it, their family, and their social life. In addition, this perspective takes into account how the new role of retiree is included in an individual’s identity and how they are able to adjust to the new role. Thus, the life course perspective highlights issues of identity development (Erikson, 1963) and identity’s dynamic nature across the life span (Erikson, 1959; Whitbourne, 1986).

### Retirement

Retirement is generally recognized as a highly individualized experience that includes myriad factors, both personal and situational (Atchley, 1982; Reitzes et al., 1996). There are also many definitions and conceptualizations of retirement. For example, Atchley (1982, p. 121) defines retirement in economic terms as withdrawal from income-earning employment, whereas Wang and Shi (2014, p. 211) characterize it in more psychological terms, highlighting decreased psychological commitment to the job.

Retiring earlier than expected, or perceiving the process as involuntary or unplanned, can negatively affect well-being (Dingemans & Henkens, 2015; Nordenmark & Stattin, 2009; Wang & Hesketh, 2012). Additionally, control over work-related decisions, including retirement, has been linked to higher levels of physical and psychological well-being (Heckhausen & Schulz, 1995; Quine et al., 2007; Whiston et al., 2015). The voluntary versus involuntary nature of work or retirement has also been represented in the literature through push–pull factors (Fisher et al., 2015; Shultz et al., 1998), both of which can affect decisions concerning retirement. Generally speaking, pull factors are seen as positive or voluntary, and push factors are seen as negative or involuntary; both can have consequences for well-being, particularly push factors—such as lack of choice to retire or when retirement is characterized as unplanned—can have negative consequences for well-being (Herzog et al., 1991; Newton et al., 2019; Nordenmark & Stattin, 2009). In previous research, involuntary retirement was found to be negatively related to life satisfaction (e.g., Newton et al., 2019), whereas voluntary retirement was positively related to well-being (Quine et al., 2007).

Thus, retirement is associated with individual differences in levels of choice and well-being, (Calvo et al., 2009; Kim & Moen, 2001). However, retirement is also associated with other individual differences, such as personal identity (Osborne, 2012).

### Identity

Identity development remains salient in later adulthood and may become particularly pertinent at the cessation of one’s working life. A personal identity often involves a commitment to a vocation and an occupational self-representation, as well as a level of inner sameness that allows individuals to navigate changes as they move through adulthood (Kroger, 2016; Whitbourne & Skultety, 2006). In one study, Kroger (2002) drew a comparison between late adulthood and adolescence, in terms of focusing on identity, finding that adults aged 65–75 were engaged in identity reintegration and re-evaluation.

Work participation can be a fundamental source of identity for many people (Calvo, 2006). For instance, Price (2000) found that in a small group of older women, many mentioned the theme of sacrificing their professional identity when they retired. As with other life transitions, retirement-related shifts in identity-defining commitments can indeed lead, as Whitbourne and Skultety (2006) observe, to identity revisions or consolidation (Lodi-Smith & Roberts, 2010; Osborne, 2009, 2012; van Solinge & Henkens, 2008). Furthermore, level of choice in whether to retire or continue working has been found to be associated with identity (Newton & Ottley, 2020), such that women who are forced to retire exhibit low levels of identity certainty.

Whitbourne (1986) developed the identity process theory as a way to understand potential identity change involved in life transitions such as retirement, proposing a three-category model for how people incorporate new information and experiences into their sense of identity as they age. The model is based on the theories of Erikson (1963) and Piaget (1975/1977). The three categories represent a relatively rigid concept of identity in the face of new information (assimilation), a relatively flexible idea of identity (accommodation), and an approach involving both maintenance and change (balance; Whitbourne et al., 2002). In terms of physical aging, individuals who use a predominantly assimilative or accommodative identity style may fare worse than those who employ a balanced approach: assimilators may continue with activities in which they are no longer physically capable; accommodators may overreact to signs of aging and feel that any preventative behavior is futile in the face of encroaching debility (Whitbourne et al., 2002). In terms of psychological well-being, Sneed and Whitbourne (2001) found that older adults who used identity assimilation and identity balance processing styles exhibited higher levels of self-esteem compared to those who used an identity accommodation process. Although not as yet tested, Whitbourne and Skultety (2006) speculate that within the context of retirement, identity assimilation—or the maintenance of a pre-retirement identity—may initially relate to better well-being outcomes. Thus, the present study examines how the three identity processes—assimilation, accommodation, and balance—relate to post-retirement well-being.

### Well-Being

In general, life events such as widowhood, unemployment, or disability can be associated with lower levels of well-being, particularly long-term well-being (Diener, Oishi, et al., 2018); however, the specific type of major life event and rate of adaptation can make a difference to long-term levels of well-being (Luhmann et al., 2012). Well-being in retirement is dependent on many factors, and while financial security and health represent two of those factors (Mackenzie et al., 2018; Topa et al., 2018), levels of post-retirement well-being can also depend on whether individuals focus on situational or personality factors (Nes et al., 2006). Psychological security, or the “…desire to stay engaged, contribute to society, and feel a sense of belonging in later life” (James et al., 2016, p. 334), might be just as or even more important. Some studies report increases in post-retirement well-being (Kim & Moen, 2001; Latif, 2011; Reitzes et al., 1996), as would be theoretically expected from the U-shaped curve (Blanchflower & Oswald, 2008; Stone et al., 2010); other studies report decreases in well-being (Bossé et al., 1987; Butterworth et al., 2006; Jaeger & Holm, 2004; Mein et al., 2003). These contrasting findings may be due to a number of factors, such as differences in methodological approach (Jaeger & Holm, 2004) and how well-being is measured. For example, using a single well-being item, Latif (2011) measured Canadian retirees’ happiness on a scale from one to five, whereas Dingemans and Henkens (2015) measured well-being in retirement using a three-item version of Diener et al.’s (1985) Satisfaction with Life Scale (SWLS).

Measuring both hedonic and eudaimonic well-being might provide a fuller picture of well-being in retirement. Although these two types of well-being show similar relationships with constructs such as gratitude and curiosity, they also have distinguishing aspects (Disabato et al., 2016), as evidenced by their definitions. Hedonic well-being has been characterized as “seeking pleasure and comfort” (Huta & Ryan, 2010, p. 735) or having high levels of positive affect and satisfaction with life (Serrat et al., 2018), whereas eudaimonic well-being has more to do with meaning and purpose in life (Serrat et al., 2018) or “seeking to use and develop the best in oneself” (Huta & Ryan, 2010, p. 735). Hence, the current study employs measures of both hedonic and eudaimonic well-being.

Consistent with the life course perspective (Elder, 1995), elapsed time since retirement has also been found to relate to well-being. For example, Kim and Moen (2002) found that men (but not women) who had retired within the preceding 2 years showed higher morale than those retired longer than 2 years; men (but not women) who had been retired for longer than 2 years exhibited higher levels of depressive symptoms. Additionally, the planned versus unplanned nature of retirement can have long-term effects: In a longitudinal study over 10 years, Dingemans and Henkens (2015) found that, compared with voluntary retirement, involuntary retirement was associated with lower life satisfaction.

### The Present Study

The present study aims to show the importance of including individual differences, retirement context, and different ways of measuring well-being in studies of post-retirement well-being. Using a sample of Canadian retirees, this study centers around this research question: How do each of the identity processes (assimilation, accommodation, and balance), the involuntary nature of retirement, and the time elapsed since retirement relate to hedonic and eudaimonic well-being?

### Hypotheses

Given previous findings concerning involuntary retirement, length of time retired, identity processes, and well-being (e.g., Dingemans & Henkens, 2015; Hershey & Henkens, 2014; Kim & Moen, 2002; Sneed & Whitbourne, 2001; Wang & Hesketh, 2012), I hypothesize that:

1. having an unplanned retirement will be negatively related to life satisfaction and meaning in life, and identity processes will be positively (balance, assimilation) and negatively (assimilation) related to both types of well-being. Additionally, unplanned retirement and identity processes will interact in their relationships with hedonic and eudaimonic well-being, such that unplanned × assimilation and unplanned × balance will relate positively to well-being, and unplanned × accommodation will relate negatively to well-being;
2. this pattern of results will also hold when time since retirement is included in analyses. Additionally, time retired will be negatively related with both types of well-being.

## Method

### Participants

Participants in the Canadians’ Retirement Expectations and Experiences study (*N* = 152) were recruited through a variety of methods (flyers posted in local libraries, businesses, and adult recreation centers; sign-up sheet at a lecture series for older adults; exercise groups) from south-western Ontario, Canada—primarily, the Kitchener-Waterloo and North York/Toronto regions. Data were collected over the fall 2016/winter 2017 period. Participants completed an online or mailed survey and an optional follow-up interview. Of the initial 152 interested participants, 136 completed the survey (89.5%). A further 12 participants had not fully retired at the time of data collection and were not included in the current study. Thus, the final analytic sample for the current study consisted of 124 retirees (*M*_age_ = 68.43; *SD* = 6.48), with a range from 55 to 86 years old. Sixty-nine percent of the sample was female.

### Measures

Responses to the specific measures outlined below were drawn from the larger survey and optional telephone interview; 124 retired individuals participated in the survey, which collected data concerning participants’ lives: family, work, personality, health and well-being, daily activities, and beliefs. Interviews were then conducted with the 102 (82.3%) participants who agreed to be interviewed. Data regarding unplanned retirement and the length of time retired were taken from the interview; unplanned retirement data were available for all interview participants; however, data regarding length of time retired were available for only 63 participants (62%).

#### Identity

The Identity and Experiences Scale (IES; Whitbourne et al., 2002) is a 33-item scale comprised of three 11-item subscales that index each of the identity processes: assimilation, accommodation, and balance. Participants rated their level of agreement from 1 (*strongly disagree*) to 7 (*strongly agree*) on each item, such as “I generally try to avoid change in my life or how I see myself” (assimilation; *M* = 4.00, *SD* = 0.81; α = .71); “I have many doubts and questions about myself” (accommodation; *M* = 2.99, *SD* = 0.98; α = .89); and “I feel confident in ‘who’ I am but am willing to learn more about myself” (balance; *M* = 5.60, *SD* = 0.59; α = .73). These internal consistencies are in line with the study of Sneed and Whitbourne’s (2003) of 173 adults (*M_age_* = 60.80): α = .72, α = .86, and α = .86, respectively.

#### Well-Being

Hedonic and eudaimonic well-being were assessed using the constructs of life satisfaction (hedonic well-being) and meaning in life (eudaimonic well-being).

##### Life Satisfaction

An overall evaluation of life satisfaction was assessed using the five-item SWLS (Diener et al., 1985). Participants rated their responses to items such as “In most ways my life is close to my ideal” from 1 (*strongly disagree*) to 7 (*strongly agree*). The mean for this sample was *M* = 5.43 (*SD* = 1.10), with α = .88, comparable to the descriptives and internal consistency exhibited in a recent study of older women (Newton et al., 2019; *M_age_*_=_ 68.99): *M* = 5.64 (*SD* = 1.09), α = .86.

##### Meaning in Life

The 14-item Meaning in Life Scale (MILS; Krause, 2004) was used in the current study. Response options ranged from 1 (*disagree strongly*) to 4 (*agree strongly*) for such items as “I have a sense of direction and purpose in life”; *M* = 3.27 (*SD* = 0.46). Internal consistency for the scale in the current study was α = .91, similar to the α = .93 that Krause (2005) obtained in a sample of adults aged 65 and over.

### Context of Retirement

Whether retirement was planned (i.e., voluntary) or unplanned (i.e., involuntary) was assessed using a single question from the interview portion of the study: “Was your retirement planned or unplanned?” to which participants responded yes or no, subsequently coded as 1 = *planned* or 0 = *unplanned*. Of the current sample, 25% characterized their retirement as unplanned.

A large percentage of those who participated in the telephone interview were directly asked “How long have you been retired?” Responses ranged from 1 year to 27 years, *M* = 7.40 (*SD* = 6.53).

### Covariates

Consistent with the life course perspective (Elder, 1985, 1995), two further covariates, health and household income, were included in analyses, along with age and context of retirement (outlined above). Health has been shown to be related with well-being over the life course (Mackenzie et al., 2018). Participants were asked to rate their overall health from 1 (*poor*) to 5 (*excellent*) in answer to the following question: “In general, how would you rate your health at the present time?” (*M* = 4.28; *SD* = 0.76). Similarly, post-retirement income is related to post-retirement well-being (Topa et al., 2018). As an indicator of income and financial security, household income was also included. Participants responded to the question “What is your yearly household income (from all sources)?” by checking responses that ranged from 1 (*under $40,000*) to 6 (*$400,000 or over*); median annual income for the current sample was between $40,000 and $100,000.

### Analysis Plan

**Figure 1:**
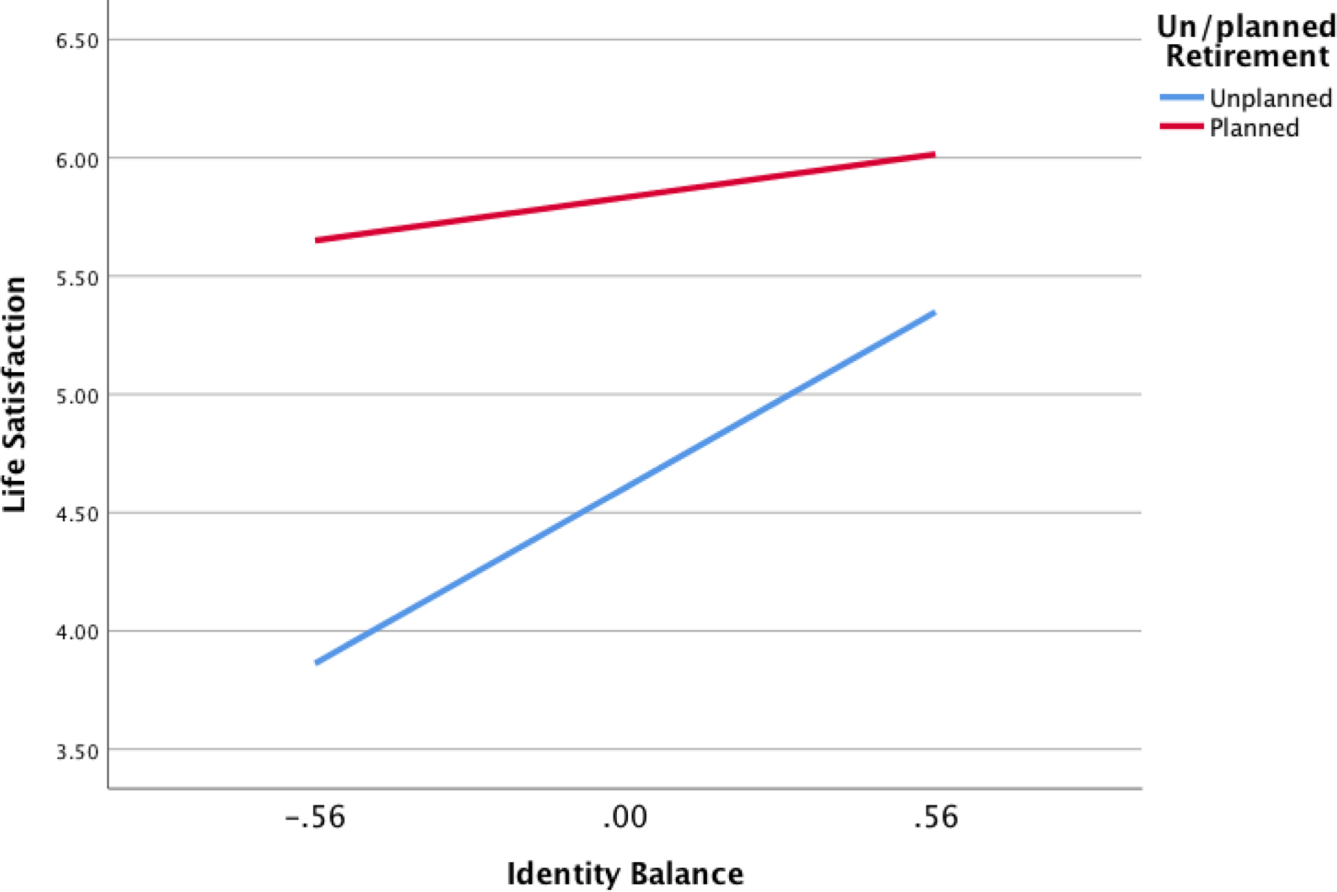
Association between identity balance and life satisfaction.

Bootstrapped correlations ascertained basic relationships between continuous variables (Table 1), followed by a series of linear regressions for the two well-being outcomes (life satisfaction and meaning in life). Due to its lack of relationship to both well-being outcomes, and in deference to the small sample size and inclusion of meaningful covariates, identity assimilation was dropped from further analyses at this point. Regression analyses examining the relationship between identity processes and well-being were conducted using the PROCESS procedure (Hayes, 2018) version 3.5.2, with retirement context included as the moderator, and age, health, and household income as covariates (Table 2). Because time since retirement can be related to subsequent post-retirement well-being (Dingemans & Henkens, 2015; Kim & Moen, 2002), and these data were only available for a subset of participants (*N* = 63), separate regression analyses were conducted to examine the same relationships between retirement context, identity process, and both types of well-being. Given the relatively strong correlation between age and time since retirement, and the small sample size, age was replaced with time since retirement for this second set of analyses (Table 3). The resultant significant moderation was further probed (Figure 1).

**Table 1:** Relationships Between Continuous Study Variables.

Variable	1	2	3	4	5	6	7	8	9
1. Age	—								
2. Health	−.17*t*	—							
3. Household income	−.25**	.23*	—						
4. Time retired	.58**	.09	−.17	—					
5. Assimilation	.09	−.09	−.09	−.03	—				
6. Accommodation	.04	−.26**	.04	−.15	−.12	—			
7. Balance	−.14	.26**	.15	.12	−.23*	−.33**	—		
8. Meaning in life	.05	.21*	.04	.14	−.04	−.59**	.58**	—	
9. Life satisfaction	−.09	.37**	.28**	.13	−.01	−.39**	.31**	.54**	—

*Note*. *N* = 116 (time retired *N* = 63); *t* < .10; *p < .05; **p < .01.

**Table 2:** The Relationship of Accommodation, Balance, and Unplanned Retirement (With Covariates) to Meaning in Life and Life Satisfaction.

Variable	Meaning in life				Life satisfaction			
	*B*	*SE B*	*B*	*SE B*	*B*	*SE B*	*B*	*SE B*
Age	.01*t*	.01	.01*t*	.01	.01	.01	.01	.02
Health	.03	.06	.03	.06	.21*t*	.12	.23*t*	.13
Household income	.04	.07	−.04	.07	.24*	.15	.15	.16
Unplanned	.06	.09	.10	.09	.45*	.20	.51*	.21
Accommodation	−.25**	.04	—	—	−.33**	.09	—	—
Unplanned × accommodation	.12	.09	—	—	.32*t*	.19	—	—
Balance	—		.42**	.07	—	—	.36*	.16
Unplanned × Balance	—		−.11	.16	—	—	−.48	.37
∆*R*²	.02		.00		.02*t*		.02	
*R^2^*	.34**		.32**		.29**		.20**	

*Note*. *N* = (116); *t* < .10; *p < .05; **p < .01.

**Table 3:** The Relationship of Accommodation, Balance, Unplanned Retirement, and Time Retired (With Covariates) to Meaning in Life and Life Satisfaction.

Variable	Meaning in life				Life satisfaction			
	*B*	*SE B*	*B*	*SE B*	*B*	*SE B*	*B*	*SE B*
Time retired	.00	.01	.00	.01	.02	.02	.02	.02
Health	−.00	.07	−.01	.07	.22	.14	.17	.14
Household income	.03	.08	−.09	.08	.19	.17	.06	.18
Unplanned	.04	.13	.26*	.12	.94**	.27	1.23**	.26
Accommodation	−.28**	.05	—	—	−.41**	.11	—	—
Unplanned × accommodation	.09	.11	—	—	.03	.21	—	—
Balance	—		.49**	.09	—	—	.52**	.19
Unplanned × Balance	—		−.23	.21	—	—	−1.00*	.46
∆*R*²	.01		.01		.00		.05*	
*R^2^*	.42**		.41**		.48**		.46**	

*Note*. *N* = 62; *p < .05; **p < .01.

## Results

Initial correlational analyses are presented in Table 1 and show that identity balance was positively related to meaning in life and life satisfaction, and identity accommodation was negatively related to both types of well-being; identity assimilation was not significantly related to either well-being measure, and exhibited no further relationship in subsequent regression analyses (and is therefore not reported further in the current study). Time retired was not significantly related to either measure of well-being. In terms of covariates, health was positively related to both well-being outcomes, whereas household income was positively related only to life satisfaction.

Tables 2 and 3 present the results from the PROCESS regression analyses, including unstandardized coefficients. Findings showed partial support for Hypothesis 1 (Table 2). Both identity accommodation and identity balance were negatively and positively (respectively) related to meaning in life and life satisfaction. The overall model for the relationship between identity accommodation and meaning in life was significant, *F*(6,91) = 7.98, *p* < .01, as was the overall model for balance and meaning in life, *F*(6,91) = 7.20, *p* < .01. However, un/planned retirement was not significant in either analysis and there were no significant interactions. In terms of life satisfaction, both overall models were again significant: for the relationship between identity accommodation and life satisfaction, *F*(6,91) = 6.08, *p* < .01; for the relationship between identity balance and life satisfaction, *F*(6,91) = 3.79, *p* < .01. Additionally, a planned retirement was positively associated with life satisfaction for analyses with either identity accommodation or balance; household income was also positively associated with life satisfaction, but only when included in the analysis with identity accommodation (no such significant relationship was found in the relationship between identity balance and life satisfaction). As with the findings for meaning in life, there were no un/planned retirement × identity process interactions. The variance associated with each of the four models ranged from 20% to 34% (Table 2).

Hypothesis 2 was also partially supported (Table 3), with results similar to those for Hypothesis 1, although time retired itself was not significant in any of the four models conducted. Both overall models examining the association between identity processes and meaning in life with time retired were significant; for identity accommodation and meaning in life, *F*(6,55) = 6.74, *p* < .01, and for identity balance and meaning in life, *F*(6,55) = 6.30, *p* < .01. In addition, planned retirement was also positively related to meaning in life, but only for identity balance, not accommodation; however, there were no interactions between un/planned retirement and identity processes in relation to meaning in life. For life satisfaction, the overall model of the relationship between identity accommodation and life satisfaction was significant, *F*(6,55) = 8.34, *p* < .01, as was the overall model for identity balance and life satisfaction, *F*(6,55) = 7.77, *p* < .01; in both cases, a planned retirement was positively related to life satisfaction. Moreover, the interaction of un/planned retirement × identity balance was statistically significant, *B* = −1.00, *p* < .05, indicating that whether retirement was planned or unplanned was a significant moderator of the relationship between identity balance and life satisfaction (Figure 1). For those whose retirement had been unplanned, the slope of the relationship between identity balance and life satisfaction was positive and significant, *B* = 1.33, *SE* = .42, *p* < .01, whereas for those who experienced a planned retirement, the slope of the relationship was also positive but nonsignificant, *B* = .33, *SE* = .21, *p* = .12. The variance associated with each of the four models ranged from 41% to 48% (Table 3).

In sum, correlations showed that identity accommodation was negatively related to both meaning in life and life satisfaction, and identity balance was positively related to both well-being measures. In regression analyses, unplanned retirement was negatively related only to life satisfaction, and only in the larger sample (i.e., without time retired included). Also in regression analyses, both identity balance and identity accommodation retained their significant relationships with meaning in life and life satisfaction; however, there were no significant interactions. When models including time retired were analyzed using, by necessity, a smaller sample, these results held, with the addition of a significant interaction between un/planned retirement and identity balance in the prediction of life satisfaction. Time retired was not related to either of the well-being outcomes.

The two short profiles that follow provide further illustration of the inherent complexity to experiencing retirement and its concomitant well-being:

“Jack” (not his real name), aged 81, is a retired teacher. He is also in excellent health, and a widower. He took an early, planned retirement, describing the process in this way:

“**…**my wife and I (who has since deceased) used to give preretirement workshops through the parks and rec**…**and we really enjoyed that but it opened my eyes to other components of retirement, such as roles and relationships, health, finance, all these things, and we made sure all those ducks were in place.”

“Shirley,” a 71-year-old widow in fairly good health, described her early retirement from teaching as unplanned; her husband retired early and was shortly thereafter diagnosed with Parkinson’s; she retired and cared for him until he died.

“We had home support workers in the house a couple of times a week to help look after him and then I was doing it and then eventually he was falling so much he progressed fairly rapidly to having to use a cane, use a walker, to a wheelchair so it was during that time that I took early retirement. He needed somebody with him all the time.”

Jack’s and Shirley’s retirement experiences are quite different. Their scores on identity processes and well-being also differ, but in unexpected ways. Given the results of the present study, one would assume that Shirley’s unplanned retirement would mean that she scored lower on life satisfaction and potentially higher on identity accommodation. Somewhat surprisingly, Shirley’s scores reflected those of someone who finds satisfaction and meaning in life, is relatively happy, and is challenged but not overwhelmed by change (i.e., she scored high on identity balance). Comparatively, she scored lower than Jack on identity accommodation; she also scored higher on meaning in life, and they scored exactly the same (6.40 out of a possible 7) on life satisfaction. Although both took early retirement, Jack had been retired for 27 years, whereas Shirley retired 16 years prior to being interviewed. These profiles demonstrate just some of the individual differences and complexities in retirement experiences, and that examining the long-term effects of retirement may be beneficial. Moreover, they underscore the issue of what to include in any study of retirement, as Wang et al. (2011) highlight in their in-depth review of retirement research.

## Discussion

This study examined relationships between retirement context, individual differences, and well-being in older Canadian adults. The first hypothesis stated that an unplanned retirement would be negatively related to well-being and that all identity processes would be related to all well-being measures in positive or negative ways; however, only two of the three identity processes (accommodation and balance) were consistently related to both well-being outcomes in the hypothesized manner. Planned retirement was also consistently and positively related to life satisfaction; there were no significant interactions for any of the four models considered in the first hypothesis. The second hypothesis stated that time retired would be negatively related to well-being, but that the same identity process associations outlined in the first hypothesis would hold; although time retired was not related to well-being, the remaining hypothesized relationships were supported, with one significant interaction (un/planned retirement × identity balance).

The results of this study are mainly in line with previous research. For example, having a balanced identity when encountering age-related change has been associated with higher levels of physical and psychological well-being, whereas identity accommodation has been associated with lower levels of well-being (Sneed & Whitbourne, 2001; Whitbourne et al., 2002). Overall, the results of examining the relationship between identity processes and post-retirement well-being suggest that having a balanced approach to incorporating new information and experiences into one’s identity in later adulthood, at least where retirement is concerned, may be more adaptive than an accommodative approach to identity processing, which entails ready acceptance of new identity-relevant information.

The current findings also suggest how individual differences can matter when assessing the relationship between identity processes and global well-being, and that the ways in which well-being is measured—whether eudaimonic or hedonic—have meaningful implications. Incorporating new identity-relevant information using a balanced approach could be viewed as more adaptive for both types of well-being, whereas using an accommodative approach to identity maintenance in later life could be viewed as nonadaptive, thus underscoring potential variability in the U-shaped trajectory of well-being during adulthood.

Although level of choice in retirement has been associated with both hedonic and eudaimonic well-being in previous research (Calvo et al., 2009; Quine et al., 2007), the present study found that a planned retirement was consistently positively related only to life satisfaction. This relationship may be unique to life satisfaction, in that when evaluating the association between societally normative (and often viewed as positive) life events such as retirement and an overall evaluation of well-being, an unplanned retirement continues to be negatively viewed, even after the passage of time. This finding also speaks to the utility of including measures of both hedonic and eudaimonic well-being in any study of older adults, both to ascertain a fuller, more accurate picture of well-being, and to account for accumulated experience of normative life transitions. In addition, while life satisfaction and meaning in life were positively and significantly correlated (*r* = .54) they do not completely overlap, thus demonstrating the related but not completely overlapping nature of eudaimonic and hedonic well-being.

The length of time since retirement may be pivotal, given previous research on the length of retirement/well-being relationship (e.g., Dingemans & Henkens, 2015; Kim & Moen, 2002). While these data were not statistically significant in the current study, future research using a larger sample that includes this information would be particularly useful in determining associations between unplanned retirement and well-being measures. It is also possible that an unplanned retirement can turn out to the retiree’s advantage, given the passage of time. Certainly, based on the findings presented here, having a balanced approach to incorporating new information into one’s identity can help one’s level of life satisfaction during retirement, particularly if retirement was unplanned. Moreover, there are myriad ways in which retirement may have been unplanned and varying degrees to which an individual can control even the most well-planned retirement process.

Perhaps individual differences or personal resources and control when one deals with change are inherently associated with eudaimonic well-being, and particularly meaning in life, whereas the context of life events—or the manner in which life transitions unfold—might be more associated with hedonic well-being, specifically in the long term (Diener, Diener, et al., 2018). In addition, one’s perceived degree of control for situational or contextual events compared with perceived degree of control of personal resources might play an important role in concomitant well-being. Future studies could examine the short- and long-term effects of retirement characteristics, differential levels of perceived control, and identity processes for hedonic and eudaimonic well-being.

### Limitations and Future Research

While informative, the present study was limited by both statistical power concerns and the unavailability of a fuller range of factors, such as longitudinal data, data concerning social support networks, or more nuanced data outlining the nature of unplanned or planned retirement. The relatively small sample size and concerns regarding lack of power preclude more complex data analysis. The sample is also well-educated, predominantly White and female, and generally healthy; these factors, combined with the sample’s geographic uniqueness (i.e., participants resided in southern Ontario), mean that it is not representative of all older Canadians. Additionally, the lack of complete data concerning length of time since retirement, and use of global and relatively generic well-being measures—not those specifically indexing well-being directly associated with the retirement experience, or more distinct indicators of the difference between hedonic and eudaimonic well-being—are also weaknesses. A further limitation is the lack of data concerning pre and post-retirement identity as well as well-being.

However, the study provides valuable insights for future research aimed at collecting these data from a larger, more diverse sample from across all provinces in Canada. One particular area in which a larger sample would be of benefit is examining the impact of work histories. For example, women’s careers often take different paths to men’s, given their traditional caregiving responsibilities; subsequently, retirement may also look very different for women (Hartmann & English, 2009; Kim & Moen, 2001), which the two short profiles provided above imply. Including data related to employment (type of work, professional or nonprofessional, full-time or part-time, continuous or discontinuous) would further illuminate the experience of retirement for both women and men.

In line with the life course perspective (Elder, 1985, 1995), ascertaining longitudinal assessments of identity processes within the context of earlier life challenges or transitions could potentially identify habitual patterns of identity revision across the life span. For example, do individuals incorporate new roles—such as spouse, parent, or widow—into their identities in the same way? Might individuals use different identity processes when faced with different roles and at different ages? Relatedly, measuring both identity and well-being before and after retirement would provide information concerning any potentially parallel change processes. Moreover, asking participants how they themselves define “retirement” might be a more appropriate way to determine a definition than merely the cessation of paid work, given the many ways in which people retire, often engaging in bridgework or un-retiring. The idea of participant-defined retirement could also extend to perceptions of planned or unplanned retirement: what does this really mean? What are the degrees of nuance between the opposing poles of planned versus unplanned?

The current study did not include a direct measure of control or control beliefs, although it could be argued that this is inherent in participants’ perception of whether retirement was planned or unplanned. To what degree do control beliefs figure in retirement decisions? Moreover, the relationship between control beliefs and identity processes may be worth consideration in future retirement research. Specifically, are primary and secondary control (Heckhausen & Schulz, 1995; Rothbaum et al., 1982) potentially related to the identity processes of assimilation and accommodation, respectively? For instance, is failing to incorporate new information about one’s experiences or context into one’s self-concept (i.e., identity assimilation) related to bringing one’s situation into alignment with one’s wishes (primary control)? In contrast, is incorporating every new piece of information and every experience one has into one’s self-concept (i.e., identity accommodation) related to bringing one’s wishes into alignment with one’s situation (secondary control)?

Finally, transitions often occur in clusters; for example, changes in health status or household structure (downsizing, change in marital status) can co-occur with retirement (Hyde & Higgs, 2004). Experiencing multiple transitions or stressors can increase the negative association of one stressor on life satisfaction (Dyrdal et al., 2019). Thus, the extent to which life transitions that co-occur with retirement are also potentially related to well-being is another avenue worth exploring.

In sum, the present study contributes to research on retirement by examining older Canadians’ eudaimonic and hedonic well-being as predicted by identity processes within the context of a planned or unplanned retirement. Findings support previous research to a certain extent, although much of the previous research has occurred outside of Canada, and the present study focused on Canadian retirees. Perhaps the most prominent finding is that having high levels of identity accommodation—or being too flexible in one’s identity—is not beneficial to older adults’ well-being, at least for the retirees in this study, suggesting how individual differences and complexity can contribute to the well-established notion of the U-shaped curve of well-being in adulthood. Consistent with previous research, however, the current study shows that retirement is a complex process and that examining both individual and contextual factors is vital to understanding retirees’ well-being.
